# The Zero Violence Brave Club: A Successful Intervention to Prevent and Address Bullying in Schools

**DOI:** 10.3389/fpsyt.2021.601424

**Published:** 2021-07-07

**Authors:** Esther Roca-Campos, Elena Duque, Oriol Ríos, Mimar Ramis-Salas

**Affiliations:** ^1^Departament of Comparative Education and Education History, University of Valencia, Valencia, Spain; ^2^Department of Theory and History of Education, University of Barcelona, Barcelona, Spain; ^3^Department of Pedagogy, Faculty of Psychology and Educational Sciences, Universitat Rovira i Virgili, Tarragona, Spain; ^4^Department of Sociology, Faculty of Economics and Business, University of Barcelona, Barcelona, Spain

**Keywords:** Zero Violence Brave Club, bullying prevention, successful educational action, friendship, mental health, bystander, break the silence

## Abstract

Bullying among peers in schools is a growing problem affecting children and adolescents from an early age worldwide. The consequences of bullying victimization in the emotional development of children and youth and their academic achievement are adverse for them and the rest of the school community, with its negative impact extending into the mid and long run. The *Zero Violence Brave Club* is implemented in schools in the framework of the Dialogic Model of Violence Prevention, a successful educational action according to the INCLUD-ED project [Strategies for inclusion and social cohesion in Europe from Education] (6th Framework Program of Research of the European Commission). The *Zero Violence Brave Club* has decreased peer bullying in schools by establishing and cultivating a culture of zero tolerance to violence in educational centers located in diverse socioeconomic and cultural contexts. This evidence-based intervention is grounded in the principle that only the person who denounces violence suffered by a peer and takes a stand always on the victim's side—and those who support her or him—against the aggressor can be considered brave. This article reports a qualitative study of the *Zero Violence Brave Club* as a successful intervention in seven schools in Spain. The schools are diverse in terms of public or private ownership, religious or lay background, and population served (different proportions of cultural minorities and students with special needs), challenging the misconception that the impact of educational interventions depends on the context. Interviews were conducted with teachers in the schools implementing the *Zero Violence Brave Club* in their class, using the communicative methodology of research. The results shed light on specific mechanisms through which the *Zero Violence Brave Club* prevents and responds to bullying in schools, such as emptying of *social attractiveness* any aggressive behaviors or attitudes. Benefits on mental health and psychological wellbeing are also reported.

## Introduction

Drawing on the conclusions from the report entitled *School Violence and Bullying* published by United Nations Educational, Scientific and Cultural Organization (1), school violence and bullying is a worldwide health problem that affects about 246 million children and young people every year. According to the report, violence in the school includes physical violence, psychological violence, sexual violence, and bullying. One of the main barriers to combating bullying is the silence of the victims of bullying (2, 3). Among the reasons for not reporting such incidents, we can find the lack of trust in adults or teachers; fear of reprisals; feelings of guilt, shame, or confusion; concerns that they will not be taken seriously; and not knowing where to seek help (1). Analyses that collected minors' voices confirmed this lack of trust in adults. For instance, in a survey conducted by the International Youth Advisory Congress (4), children affirmed that they did not share with their parents the details about the online contents they access.

Several studies have reported the consequences of bullying and school violence on children and adolescents' mental health (5), and bullying victimization and mental health seem to be associated with each other among adolescents (6). Being bullied has been associated with anxiety and depression symptoms (7), with having more severe mental health problems (8), and with an increased risk of suicidal ideation (9). Moreover, the risk of psychotic-like experiences in adolescents may be increased by the number of traumatic events experienced—including bullying (10). Wu et al. (11) focused on the consequences of bullying on mental health depending on the role and found that these consequences are more important for victims and bystanders than for bullies. Remarkably, while defending behaviors were positively associated with social anxiety in bystanders and with depressive symptoms in both victims and bystanders, they were not significantly related to mental health indicators in bullies. It is essential to study the impact of bullying on mental health not only because of the reduced wellbeing of children and adolescents during the period bullying occurs but also because the early onset of mental health problems can be a risk for the subsequent development of psychiatric disorders in adulthood (10).

### Educational Interventions to Prevent and Eradicate Bullying

Educational interventions have been implemented worldwide to prevent and eradicate bullying, and research has analyzed the effects of implementing specific anti-bullying policies and programs. The *Olweus Bullying Prevention Program* (OBPP) was created by Olweus (12), one of the most relevant authors in bullying prevention. This programme pays particular attention to including bullying in the design of curriculum activities and teacher and family education. A study evaluating the program's effect in Norway and the United States (13) reported a reduction in bullied students after implementing OBPP in schools.

KiVA is another programme being evaluated widely. This programme was initiated by a group of researchers from the University of Turku and supported by the Ministry of Education of Finland. KiVA is similar to OBPP, but it places more emphasis on the role of peers in the identification and stopping of bullying. Kärnä et al. (14) carried out an extensive evaluation of the programme in a sample of 78 schools, and the findings demonstrated the effectiveness of KiVA in reducing bullying and victimization. Furthermore, KiVA increased anti-bullying behaviors in classrooms, indicating students' awareness of the need to protect victims. The studies report that the KiVA programme reduces the prevalence of harassment and victimization. For instance, in the case of primary education, data shows a reduction of 30% in self-reported victimization and a decrease of 17% in self-reported harassment, compared to control schools.

Besides the analyses that focus on examining the reduction of bullying rates, other effects have been identified. Ttofi and Farrington (15) conducted a meta-analysis on the effectiveness of different anti-bullying programs in schools published between 1983 and 2009. One of its major conclusions was the connection between school climate and academic achievement. In this regard, a study conducted in the United States reported that low rates of bullying are linked with high graduation rates 4 years later, suggesting that, when bullying is predominant in schools, dropout rates are above the state average, while schools with low levels of bullying exhibit a radical decrease in dropouts (16). This is consistent with research that has emphasized that the improvement of coexistence among the students and the educational community and academic learning are closely linked. Educational strategies are more effective when they take this link into account (17).

Other studies pay attention to the impact of anti-bullying interventions on children's mental health or those aimed at improving psychological wellbeing when coping with bullying. For instance, Moore et al. (18), who conducted a controlled trial with 283 students in different Australian schools, found that a strengths-based psychosocial intervention improved children's health status, particularly in terms of resilience and self-efficacy. In the same vein, the investigation carried out by Guimond et al. (19) showed that schools where anti-bullying policies are promoted and where teachers perceived themselves as efficient in handling bullying situations were more protective for youth at risk of developing anxiety problems and developed fewer symptoms. In this regard, an exhaustive study on the effects of educational policies in the United States with an inclusive approach in the reduction of suicide among Lesbian, Gay, Bisexual, Transgender, and Intersexual (LGBTI) students experiencing bullying at school showed that schools that followed these policies presented lower percentages of risk of suicide than did those which did not implement them (20).

### Bullying and School Climate

Along with the effects, research on bullying and the related intervention programs has discovered a set of effective strategies in preventing and eradicating bullying. Mayes et al. (2) reported the following strategies: fostering strategic leadership, creating safe environments, promoting mechanisms to enable victims to report incidents, and providing permanent support to victims. Cornell et al. (16) summarized the following five types of interventions as effective strategies: (a) establish a shared vision with the entire school community about the type of school they would like to be in, (b) evaluate the school's strong points and needs from a comprehensive perspective; (c) include prosocial skills in the curriculum; (d) involve students in developing preventive strategies; and (e) promote partnership between families, teachers, and other professionals to promote bystanders' ability to stop bullying. Both analyses highlighted the importance of working with the educational community, incorporating the leadership between peers to prevent school bullying. It has been considered a fundamental value among peers for prevention programs by significantly correlating with reducing both school harassment and victimization (2, 16).

Studies on the effects of interventions that take into account the role of diverse agents in the school have reported a reduction in rates of bullying and victimization and, at the same time, an improvement in academic achievement (21, 22). Overall, studies agree that effective interventions based on comprehensive approaches can reduce this phenomenon (23, 24). Midthassel et al. (25) highlighted the central role that principals had in implementing a public programme to reduce bullying in Norwegian schools, showing the relevance of consolidating firm leaderships that strengthen this strategy. Recently, more research has focused on the effectiveness of two of the abovementioned strategies: those linked to community involvement (26) and those related to being an active bystander (27). Concerning the first strategy, investigations have revealed that programs where regular meetings are organized with families to discuss norms against bullying have achieved good results in improving school climate (28, 29). In this sense, family and community involvement through decisive and educative participation is related to improved coexistence (19). *Decisive participation* is characterized by families and other members of the educational community in the decision-making processes to actively decide what kind of norms should be respected in the school, allowing all stakeholders to internalize and appreciate them and reduce coexistence problems quickly. *Educative participation* also directly influences school climate because parents and other community members are present in different educational spaces, helping teachers build a positive coexistence, for instance, inside the classroom by supporting children's schoolwork and supervising whether the agreed norms are respected.

Studies on the effects of bystander interventions have revealed the critical role of bystanders in protecting victims. Several analyses have reported that peer-to-peer interactions are crucial for preventing bullying and reducing victimization rates (30, 31). Prevention of bullying based on the research about bystander intervention is linked to the idea of being an active rather than a passive observer of bullying or any other type of violence, that is, to not being an accomplice of bullying–victim behaviors. Contrarily, it implies the ability to say no to this kind of interaction and stop any aggression. Literature on this topic calls such active bystanders as upstanders, who have been found to have a significant influence on prevention programs (32). Besides, studies on the effects of bystander intervention demonstrate that the support received from teachers and other school staff is crucial to spur bystander actions (33). Research has also analyzed the peer status associated with the bullying role. The study conducted by Guy et al. (34) showed that bullies received the highest scores in perceived popularity. Victims and bully victims scored lowest in social preference, suggesting that bullies receive peer reward for their behavior. This is crucial for anti-bullying programs that rely on peers as active bystanders.

### Theoretical Framework of the Zero Violence Brave Club

The present study aims to gather evidence on bullying prevention and reduction in schools due to implementing a specific strategy, called the *Zero Violence Brave Club* (35). This strategy is based on a successful educational action called Dialogic Model of Violence Prevention (DMVP) which was evaluated as a part of the project *INCLUD-ED. Strategies for inclusion and social cohesion in Europe from education*, funded by the 6th Framework Program of the European Commission.

Research has shown that the DMVP contributed to improving coexistence and reducing bullying due to fostering deliberative processes in defining the school's rules among the different agents, including families, teachers, and pupils (36, 37). The case studies about the implementation of the DMVP are schools that highlight its impact in preventing school violence. It can be seen in the increase of the empowerment of the students to reject and give visibility to the existing violence (the number of reports increase) and in the creation of support and friendship networks among the community to achieve schools of zero tolerance to violence (38, 39).

There are three main characteristics of DMVP: (a) agreement on the school's rules based on the participation of the whole community (families, students, teachers, and other social agents), (b) zero tolerance of any violent behavior, and (c) implementation of strategies that promote preventive socialization of peer-to-peer violence. The leadership of all the students in peer prevention is a critical factor in creating a safe environment free of violence (40). This third characteristic is based on the assumption that children and adults are used to socializing in spaces where violence is normalized or even promoted. To combat this dynamic, it is necessary to apply interventions that add value to alternative behaviors and make kindness attractive for children.

The *Zero Violence Brave Club* programme is one such example. It is based on how students take a stance against violence and report it whenever it occurs, while they place value on friendship. In this club, students learn to defend and support victims and to isolate students who behave violently. The *Zero Violence Brave Club* programme opens up a space of dialogic leadership in which students share daily situations and conflicts about feelings and values, where the ethos shows a clear positioning for eradicating violence.

The programme was developed in 2014 in different schools—both public and private—in pre-primary, primary, and secondary education, and it is grounded on the theory of the preventive socialization of gender violence developed by Gómez (41) and led internationally by CREA (Community of Research on Excellence for All) (42).

The *Zero Violence Brave Club* is grounded onto four key aspects: (1) the relevance of the socialization into the attraction toward nonviolent models of relations; (2) the importance of implementing zero tolerance to violence from zero years of age and of involving the whole community in the stance against violence; (3) the dialogue and leadership of the students in peer prevention as a critical factor for the creation of a safe environment free of violence; and (4) the training with the teachers and the community into the scientific evidence of the violence prevention (35, 43).

The Zero Violence Brave Club in schools is operationalized as groups of children with nonviolent values. They support any child in their class or school who wants to avoid the aggressions they receive from other children in the school, acting as a peaceful shield against the aggressors (44). They are called brave because they work respectfully and without violence, and one cannot be part of the club if having violent behavior. Once the bullies stop such actions, they are reintegrated into the club (35). Therefore, the *Zero Violence Brave Club* encourages peers to be active bystanders and has a crucial component in the promotion of interactions that empty aggressive behaviors and attitudes of any social appeal. The ones who best know what happens to the students in the classrooms and the playgrounds are the very students. This can only be achieved through a joint effort around the *language of ethics* and the *language of desire*, showing that what is desired and attractive can be good and nonviolent (45).

The permanence of the Zero Violence Brave Club as part of the centre's daily life and not as a one-off activity makes it possible to work on these interactions in-depth and, through dialogue between peers, to transform the desire associated with violence (46).

Another of the keys included in the *Zero Violence Brave Club* is protecting both the victims (first-order) and the people who defend these victims. In so doing, children learn to prevent what is internationally known as second-order sexual harassment from an early age. This is known as physical and psychological violence against people who support victims of violence (47). Overlooking these measures often leads witnesses to consider not intervening in support of the victims for fear of being victimized, i.e., fear of being a future target of bullying (48).

Focusing on the *Zero Violence Brave Club* program, this investigation contributes to building new knowledge on bullying prevention or anti-bullying programs because it provides novel elements concerning preventive efforts against peer-to-peer violence.

## Materials and Methods

This study is developed following the Communicative Methodology of Research (CMR) (49), characterized by the inclusion of egalitarian dialogue among the participating agents throughout the research process. In this research, teachers leading the implementation of the Zero Violence Brave Club in the schools under study were interviewed (50). In the CMR, dialogues between researchers and research participants are based on validity claims instead of power claims. Such interactions enable researchers and end users to interpret social reality dialogically and create knowledge to transform inequalities (51). In the present study, the CMR allowed researchers and teachers involved in the *Zero Violence Brave Club* to engage in an egalitarian dialogue around two key issues: the impact of the programme on reducing and preventing bullying and the components within the programme that make this possible, improving coexistence and children's wellbeing.

### Study Design

The objective of this study was threefold:

1. to collect qualitative evidence of improvement in coexistence and bullying prevention and reduction as a result of the implementation of the *Zero Violence Brave Club*;
2. to analyze the components of the *Zero Violence Brave Club* that contribute to bullying prevention and reduction; and
3. to analyze the impact of the *Zero Violence Brave Club* on children's mental health and psychological wellbeing.

For this purpose, the schools were selected by convenience sampling. A sample of seven purposively sampled schools was selected to collect the data. The chosen schools had to meet the following criteria: (1) schools implementing the Zero Violence Brave Club in at least one classroom during 2 years (In the first meeting to present the study, the teachers assessed that 2 years was the minimum time they needed to implement the programme and observe and collect results); (2) schools which are diverse in terms of socioeconomic status of its students, size, location (urban and rural), ownership (public or private), religious or lay background, and population served (different proportions of cultural minorities and students with special needs); and (3) schools involved in the Seminar called “On the Shoulders of Giants” in Valencia (52), a seminar in which teachers receive training on bullying prevention based on scientific evidence. Attendance at the seminar ensured that the teaching staff knew in depth the implementation criteria of the Zero Violence Brave Club.

Seven schools were selected in Valencia (Spain), which had been implementing the *Zero Violence Brave Club* in their classrooms for 2 and 3 years. Table 1 presents a summary of the characteristics of each school where pseudonyms have been used to ensure anonymity.

**Table 1:** Characteristics of the schools included in the present study.

**School**	**Characteristics**
School 1	It is a private school based on a religious tradition located in Valencia (almost 800,000 inhabitants). It offers the following levels of education: primary, secondary, postsecondary, and vocational training.
School 2	It is a public school that offers pre-primary and primary education. It is located in a small town with 21,600 inhabitants. It has around 450 students, with families from the area.
School 3	It is a public school that offers pre-primary and primary education. It is located in a small town comprising 25,000 inhabitants. The school has 508 registered students, including a small number of migrant students.
School 4	It is a public school that offers pre-primary and primary education. It is located in a town comprising 23,000 inhabitants. It has a long history in the city and currently has 300 students.
School 5	It is a public school that offers pre-primary and primary education. It is located in a large town with 74,000 inhabitants. It currently has around 450 students. Most families are from the area, but there are migrants as well.
School 6	It is a school with a Catholic tradition sponsored by the Catholic Church and Public Bodies. It is located in Valencia. It offers different levels of education, including pre-primary, primary, secondary, postsecondary, and vocational training. It has a 98% of Roma students.
School 7	It is a public school addressing special needs. It offers pre-primary, primary, and secondary education and courses to aid the transition to adult life (for 16- to 21-year-olds). It is located in a small town with 8,200 inhabitants. It has around 240 students.

The data collection was organized through structured online interviews with teachers. This instrument differs from an open-ended questionnaire because the interaction between respondent and interviewer continued after the respondent answered the questions. Thereby, exchanges can be synchronous—happening in real time-, or asynchronous—days or weeks after the questions were asked (53). In this study, we followed the second strategy (asynchronous). All the respondents answered the same open-ended questions, which were presented in a word file. We sent this file to the teachers by email, explaining the study's objectives. Two weeks later, we contacted the teachers again to provide follow-up information that allowed the creation of intersubjective knowledge creation, a distinctive feature of the communicative methodology.

**Figure 1:**
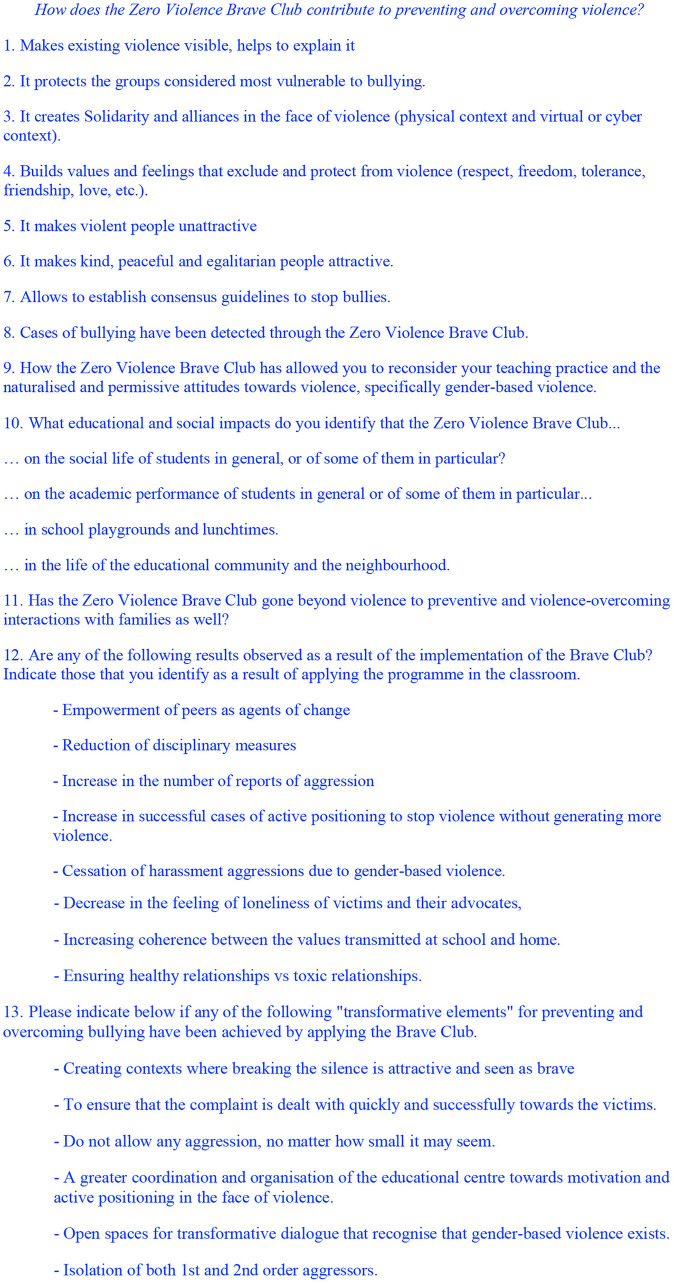
Online interview outline with teachers who apply Zero Violence Brave Club at school with children.

A total of 10 teachers from all schools participated in the research (four men and six women). The teachers who participated in the study had been developing the *Zero Violence Brave Club* in their schools for 2 or 3 years and could inform about children's interactions and dialogues and the effects of this strategy on their pupils. They were teaching in different educational levels, namely: primary and secondary education, and training courses addressed to students with special needs, to facilitate the transition to adult life. Therefore, the age of the students they worked with ranged from 6 to 21 years. All the teachers answered all the interview items except one teacher who did not answer items 10, 12, and 13 of the interviews' outline because of a lack of experience in implementing the programme due to being new at the school (see Figure 1).

For the design of the interview guidelines, we first reviewed the literature to identify common elements that characterize effective interventions against bullying. Second, we discussed with teachers involved in the research about the features that characterize the *Zero Violence Brave Club* (see Figure 1). This procedure allowed us to design the outline for the interview considering end users' voices. The conversations with teachers also informed about the school's climate prior to implementing the *Zero Violence Brave Club*, which was also informative of the prevalence of violence and actions to address it. For instance, in School-7, peer-to-peer violence occurred day after day. Similar situations were reported in the School-4 where, before implementing the *Zero Violence Brave Club*, violence was normalized, and it was very usual to see mockeries and fights inside the school. In most of these schools, like School-3 or in School-6, staff had previously applied a disciplinary model of conflict resolution. This strategy did not change the school's climate, and there were schools—like School-6—with more than 60 disciplinary reports in 1 year. In School-1, teachers confirmed that conflict situations had not been handled in the past and, therefore, they persisted over time.

### Data Analysis

The data analysis sought to identify how the *Zero Violence Brave Club* contributed to preventing and reducing bullying in school classrooms. For this purpose, an analytical grid was created, with three categories of analysis and two dimensions developed in an inductive–deductive process, considering both the main topics emerging from the data collected and the relevant contributions from the literature. Firstly, we established a group of categories based on the information collected from the online structured interviews. Secondly, we contrasted these categories with previous knowledge about the Zero Violence Brave Club (35) and other investigations that had analyzed strategies against bullying and allowed to pinpoint a set of analytical elements highlighted by research as being essential to study bullying. Two researchers led the process of coding and induction of the themes. One of the researchers from Universitat Rovira i Virgili carried out the first phase of emptying and classifying the information by category of analysis. Subsequently, a second review of the categorization of the data was carried out by a second researcher from the Universitat de Valencia. The coding consisted of identifying the data provided by the participants. This part of the study consisted of coding the transforming elements detected by the teaching staff into each of the three categories of the analysis presented below. Given the size of the sample and the type of information provided by the teachers (much of it in the form of narratives), the use of an analysis programme to identify the data was considered unnecessary. This information was conveniently recorded according to the analysis matrix setup.

Table 2 shows the analytical grid composed of the three categories and two dimensions. The coding criteria were agreed upon in two meetings of the research team. We discussed how to code each of the quotes extracted from the teachers' interviews assigning the appropriate number from the analytical grid. Following the premises of the CMR, for each of these categories two dimensions were considered: exclusionary which refers to the barriers that prevent individuals from enjoying a social benefit or a right, and transformative which concern the actions contributing to overcome the barriers (51). In the present study, the exclusionary dimensions were social barriers that reproduced bullying in educational contexts. The transformative dimensions focused on the *Zero Violence Brave Club's* effects on preventing and overcoming bullying. In this article, we focus on the findings around the transformative dimensions to identify effective elements among the interventions addressing bullying. Exclusionary factors are also indicated, illustrating the difficulties and controversial aspects that come up in schools when the *Zero Violence Brave Club* is implemented.

**Table 2:** Analytical grid.

	**Breaking the silence**	**Promoting real friendship**	**Making violence less attractive**
Exclusionary dimensions	1	2	3
Transformative dimensions	4	5	6

The definition of these categories is as follows:

1. *Shedding light on the existing violence contributes to breaking the silence*
  This category refers to how the *Zero Violence Brave Club* contributes to creating awareness regarding bullying. Research on gender-based violence and peer-to-peer violence illustrates how the public denounces aggression or harassment in private or public spaces, preventing further attacks (54, 55). This is a reality that also emerges in classes where the *Zero Violence Brave Club* is implemented.
2. *Promoting real friendship that protects from violence*.
  This category is related to how the *Zero Violence Brave Club* creates feelings of solidarity and friendship that protect from bullying. Analyses on peer-to-peer interactions in the classrooms exemplify friendship's relevance to preventing bullying practices (56). In the *Zero Violence Brave Club*, this element appears because children develop positive feelings with victims; they want to protect them and become friends.
3. *Making violence less attractive*.
  This category refers to the effect of the *Zero Violence Brave Club* on making bullies less attractive and giving visibility and greater appeal to brave (nonviolent) children. According to previous studies on preventive socialization, there is a coercive discourse that links violence with attractiveness (57). In the *Zero Violence Brave Club*, this dynamic is combated through peer-to-peer interactions and teacher-to-pupil interactions, through which violence is made less attractive.

### Ethics

This study draws on the Universal Declaration of Human Rights formulated by UNESCO, the Charter of Fundamental Rights of the European Union, and the Council of Europe Strategy on the Child's Rights (58). Participants were provided informed consents that included complete information about the purpose of the study. Research data was safely stored in the computers to which only members of the research team have access. Confidentiality was guaranteed because the names of the respondents do not appear in the files used for the analysis. The real names of the schools have been changed to pseudonyms, and the data collected from the participants has been anonymized. The study was fully approved by the Ethics Board of the Community of Researchers on Excellence for All (CREA)^1^.

### Data Statement

Our study has been registered with the Open Science Framework under the following link: https://osf.io/49bpk/.

## Results

The evidence collected from the teachers' experience indicates that the *Zero Violence Brave Club* has contributed to reducing bullying in the classes where it has been implemented. The general perception of adults in the school (teachers, educators, or other professionals in the schools and families) is that conflict has been reduced. Although violence has not entirely disappeared, the severity of the cases has been reduced within and outside the classroom.

There has been a substantial improvement in the playground and the school canteen with those groups where I have implemented the Brave Club. The general comment of other adults is that there has been an improvement and that there is less conflict and [when it happens, it is] less severe. The reality is that conflict has continued to occur, but more as a lack of respect and less severe physical or verbal aggression [Teacher 2—School 2].

According to teachers' perception, the programme had a crucial role in changing the dynamics and it is now more frequent to see children talk to each other, learning from one another about the best way to solve their problems. The effectiveness of this approach against bullying in some cases has led the school to train other staff to guarantee a coherent intervention in the different spaces of the school:

The classes that implement the Brave Club spread it to others whom we see running to look for the help of an adult explaining what happened to them. I have noticed that there are fewer situations and, especially, that they are less severe. Physical aggressions that happened in the playground have disappeared, and now what still happens is that they get angry when they play, or they improperly speak to each other. They bring along the conflict to the school canteen, so it is important to explain it [the programme] to the school canteen assistants because kids notice the possible contradictions [occurring between norms in the playground and the school canteen]. This year we gave training to the assistants [Teacher 5—School 4].

Most of the interviewed teachers agree that the reporting for peer aggression has increased and the number of cases in which peers have taken an active part in stopping violence. Peer empowerment is being perceived as a critical agent for change. Many teachers also agree that they have overcome the trivialization of violence, and no aggression is allowed, regardless of its severity. Many of these schools have managed to guarantee a quick and effective response in front of a call for help or a complaint, and teachers believe that their students have now healthier and less toxic relationships. In the following sections, the main components of the *Zero Violence Brave Club* that have contributed to these improvements are analyzed.

### Shedding Light on the Existing Violence Contributes to Breaking the Silence

The implementation of the *Zero Violence Brave Club* in the classroom opened spaces of dialogue in which victims felt safe and comfortable to explain any negative situation they had experienced at school or in any other educational context. We believe more instances of bullying emerged in the schools because students tended to make it more visible. Even bullying cases that had been occurring for years were now revealed with the *Zero Violence Brave Club*:

The implementation of the *Zero Violence Brave Club* in the second year of primary education in the school year 2015–2016 facilitated that a case of school bullying that some girls in the class were doing to another girl—and which had started 2 years earlier-, was made visible. This girl had serious problems, both academically and socially. The fact of reporting worked as a stimulus for her, mainly at the academic level. (…) the girl started to improve her academic performance rapidly [Teacher 3—School 2].

One teacher, who was also the principal in one of the participant schools and had implemented the *Zero Violence Brave Club* with children aged 6–7, explained that students observed this intervention as a space to talk about unfair situations. In their class, children felt empowered to explain such conflicts because, within that strategy, children internalized the idea they are “brave” for sharing these experiences. The following quote exemplifies this reality from a teacher's reproduction of a child's words:

In the beginning, I was reluctant…., but since I know what a brave person is, I speak up about the injustices because, otherwise, the one who does them, the coward, wins [Teacher 6—School 4].

As a part of the creation of an atmosphere where zero violence is promoted, it was common to hear dialogues where children explained their experiences when facing aggression, showing how this atmosphere empowered them to talk about these episodes and to meet them, such as one of the teachers reproduces in the following example:

A. pulled my hair; he is a coward, and I will not allow him to treat me this way [Teacher 9—School 7].

Teachers described that students reported feeling safe to make comments like this, thanks to these dialogic environments. The fact that children dare to talk about their experiences of bullying is connected to the certainty that they will have the support of the class. Then, fear turns into confidence, as a teacher explained:

Reports are multiplied and so is the action of taking a stance when there has been a conflict in the different spaces of the school. When the students enter the classroom after recess, they claim to be able to make public what happened in the playground, whether it is about their classmates or about children in other class groups. (…) In the class assembly, children explain what happened, the attitudes each child showed, and they relate it to friendship and the idea of zero violence. Children who are victims dare to explain what happened to them because they know that they will have the support of their class. (…) Recently, a boy from class B, with the support of other boys of the class, dared to report that an older boy from sixth grade had attacked him. Although he was still afraid of the consequences, when he saw that the entire classroom gave their support to him and told him that they would not leave him alone, he was touched [Teacher 2—School 2].

Thus, following the insights of one of the teachers interviewed, the *Zero Violence Brave Club* enabled students to learn to identify more situations and encouraged them to report such incidents to any adult: “Before we didn't discuss (…) and since we have started the Brave Club, we explain aggressions.”

According to teachers' experience, peer support is a crucial component of the *Zero Violence Brave Club*, which powerfully sheds light on the existing violence in the schools. The *Zero Violence Brave Club* is characterized by an explicit display of support from the whole community and, particularly, from peers to victims. This dynamic contributed to breaking the silence about the existing violence. The following quote of a teacher explaining what happened with a humiliated student in the playground exemplifies this effect. After this tough episode, the student openly reported the bullying in class; the supporting climate created in the *Zero Violence Brave Club* encouraged him to share the experience and, after explaining it, he received the peers' support again:

Once, during recess, a student felt humiliated by another: the other child told him that he did not know how to play football and that he had to “eat the grass.” He told the adults overseeing the playground, but their recrimination to the other student did not help him. Later, in the classroom, in his *Zero Violence Brave Club*, he expressed the need to share it with his tutor and classmates. He told us how he felt bad and cried because he did not understand why the other student had said those things. His peers empathized with him, showed him their support, and encouraged him to continue playing football because they knew he was a good player. Everyone expressed that the other student's behavior was not good, and they supported their friend [Teacher 8—School 7].

Not only did the students' response change in front of violence, but the community perception of violence also changed as a result of implementing the *Zero Violence Brave Club*. Teachers expressed their satisfaction when families supported them to manage daily life situations at these schools, and they felt comfortable working together to ensure good coexistence between children. Opening dialogue spaces in the school extends these reflections to other members of the educational community. One of the teachers involved in the research explained a conversation with a mother, in which she stressed the importance the school gives to any conflict situation occurring in the classrooms or any other spaces. This mother felt that sometimes adults perceive children's violence as insignificant and emphasized that it is crucial that the school helped not to normalize this violence. The role of teachers was also revealed as capital to promote a violence-free school context and a climate where it is safe to talk about the violence children face. When this role was shared with the community and with the students, teachers became more aware of the importance their role had to promote this process of breaking the silence. In the following quote, a male teacher explains an episode when he reinforced a victim who talked about a bullying situation. Calling him “brave” for telling the truth not only reinforced the child's attitude in this situation but also promoted it to be sustained in the future.

Teachers reinforced behaviors through verbalizations when they were listening to the complaints of students and fostering help from peers, such as: “how cool it is, to tell the truth, you are very brave, we will support you, count on whatever you need, you have a friend who is very cool because he loves you and helps you, we are going to tell other classes so they will be able to see how brave you are.” This way, the aggressor is not supported by anyone, his/her appeal is reduced, and the bully realizes that something doesn't work, and it needs to be changed [Teacher 9—School 7].

### Promoting Friendship That Protects From Violence

Evidence collected from teachers illustrated the process of shaping a set of attitudes, feelings, and interpersonal bonds that offer protection against bullying. First, teachers argued that the *Zero Violence Brave Club* had become a learning space in which children understand the meaning of respect for others in practice. This was observed, for instance, by a parent that reported to one of the teachers participating in the study that some students who were disrespectful before implementing the *Zero Violence Brave Club* changed after its implementation. They discovered the meaning of being respectful by practicing supportive behaviors. A teacher explained what a mother shared with her about her 9-year-old son:

I think that indeed P. has changed 100%. Like they picked on him at the other school, and when this happened he got into fights. He came here and discovered respect, the *Zero Violence Brave Club,…* it took him a while, but [now] he understands what is right, what is wrong and if he sees his sister laughing at someone, he goes and tells her: you shouldn't do that because that's not for the brave, that's for cowards, you have to respect, you have to have respect. I think he has significantly changed in that, above all, in the respect for others [Teacher 5—School 3].

Second, teachers have also observed that the *Zero Violence Brave Club* promotes feelings and attitudes of solidarity. Many teachers report that the feeling of loneliness among victims and those that defend them has been reduced. These are cases in which solidarity has been shaped to protect those peers who felt intimidated or fearful so that they can participate in the group activities now, feeling safer.

With the club, students build alliances for protection. The other day, a girl was feeling intimidated by another girl from another class because this girl bothered her all the time in the playground. A group of the class told her to join them in the playground so that she felt protected [Teacher 1—School 1].

According to teachers, the *Zero Violence Brave Club* fostered group awareness, which generated respectful behaviors among pupils despite their differences and supportive behaviors when disrespectful or violent behaviors occurred. Solidarity, which is built on respect and goes beyond it, has created new opportunities to develop friendships. A teacher from one of the schools shared some examples of this reality:

The Braves' Club has allowed creating the group's awareness where, despite the differences among students, all are respected (…), and now they enjoy being together. For instance, in the last year, a student with Autism Disorders Spectrum did not share games or activities with others. He preferred to stay alone (…). At the end of the year, he requested to play with a specific classmate. He accepted that he would help him; he became his friend; and although he did not realize when someone made fun of him, his friend defended him [Teacher 8—School 7].

This solidarity has often been translated into peer support that has made it possible to break the silence about bullying situations at present or lived in the past. A teacher confirmed this effect of solidarity by narrating a girl's testimony in the *Zero Violence Brave Club*, explaining the terrible harassment she had lived. When her peers listened to her experience, they started supporting her and acting as upstanders. Until that moment, episodes of bullying had been silenced while they were known; from the moment children were able to discuss this and other violent situations in the framework of the *Zero Violence Brave Club* and thus develop solidarity, reports of bullying increased in the school:

Three years ago, in the fifth grade, in one of the first sessions of the Braves Club (…) a girl dared to explain that, in fourth grade, a classmate had tied her to a lamppost in the playground with a string and that another child had been forced to kiss her while she was tied. Nobody in the group had reported it, although many of them knew what happened. After the dialogues that emerged in the Brave Club, they became aware of the gravity of the situation, and they began to support the victim. For some time, that child [the bully] had no one to play with on the playground. From that moment on, complaints about disrespect or violent attitudes increased [Teacher 2—School 2].

A third issue that emerged in the teachers' narratives was the role of the *Zero Violence Brave Club* in teaching children about their freedom to choose the people with whom they want to relate, and particularly how to choose their friends. Students in the club learned to identify peers who will treat them properly or badly and select their friends accordingly. The following quote of a teacher explains this dynamic by describing the dialogues she established in the classroom to help students internalize who is respectful and *brave*, or disrespectful and a *coward*.

In class, we talked a lot that friends always treat us well and that those who do not treat us well are left without friends; everyone has freedom of choice to decide how they want to treat or interact with others and choose people who always will treat them well. One day, a boy said that the key to being in the Brave Club was to treat others well, and when someone didn't treat someone well, he was reminded that he had lost “the key.” This helped to start dialogues about how to choose freely. The club also allows them to say without coercion or pressure with whom they want to be or play, always based on the criterion of being treated well [Teacher 5—School 4].

Teachers were also positive about the fact that the context created by the *Zero Violence Brave Club* provided children with the opportunity to decide about their classroom's rules, and always link these to nonviolence. This context also positively influenced the children's process of learning to decide who they wanted to be friends with. For instance, as shown in the following quote, a primary education teacher explained how a rule agreed in the community—“I like you to treat me well”—helps children to choose their friendships freely and based on respect and care for others:

The *Zero Violence Brave Club* promotes alternative behaviors that can stop violent ones. Our school has the rule, “I like you to treat me well.” So, alternative behaviors [when violence occurs] such as “I don't care about you” or “I do not like that” allow the group to transform itself, rejecting violent behaviors and achieving a healthy environment for work and cohesion in the classroom [Teacher 7—School 6].

These attitudes and feelings were socialized in children's interactions during their participation in the *Zero Violence Brave Club* and were present in their daily experience. A teacher shared the following examples from their primary school students' interactions: “We all have the right to freedom; we should respect others” and “nobody can tell us whom we should go with.”

Despite these transformative elements that become evident when the *Zero Violence Brave Club* is analyzed, teachers also highlighted that it is not a smooth process and that some barriers exist that make respect and solidarity difficult. For instance, they reflected on how children reproduce aggressive behaviors that are usually normalized in different social contexts. Children imitate the behaviors they experience in the street or at home. A clear example is a typical sentence, “if they hit me, I hit back.” This normalization of violence led to some kids acting selfishly and disruptively during the classroom assemblies making noise or acting aggressively, interfering with the group's dialogue. Teachers stopped these behaviors and temporarily separated these students from the group, thereby using these situations to teach their students that violent attitudes would not be allowed, countering the normalization of violence and promoting alternative socialization.

### Making Violence Less Attractive

Most of the teachers who participated in this study talked about attractiveness as a critical aspect in the interactions promoted within the *Zero Violence Brave Club*. They argued that interactions and dialogues enabled in the club affected children's interests and tastes and changed what they perceived as attractive. With the implementation of the *Zero Violence Brave Club*, students learned to dislike children who engaged in violent behaviors, and they preferred to spend time and have fun with peers who treated them well. Many teachers also inform that the *Zero Violence Brave Club* has enabled to build contexts where breaking the silence is also perceived as attractive. This is how the bullies lost the prominent role they used to have in the classroom, and therefore their behavior was not reinforced.

In other cases, there is a significant reduction in the appeal of those who seek social attractiveness through showing negative attitudes and behaviors. The fact of publicly exposing violent attitudes and valuing positive attitudes of solidarity takes the spotlight off people who are not “brave”; they become less visible. [Teacher 2—School 2].

Teachers stated that, following the implementation of the *Zero Violence Brave Club*, the children did not perceive violence as attractive, and they rejected violence in different daily spaces inside and outside the school. For instance, a teacher explained a dialogue he had with a mother about her daughter's rejection of going to the park due to the presence of a girl who was constantly making fun of others and bullying her. She preferred to go elsewhere, showing her perception of spending her time at the park as a non-appealing activity. According to teachers' reflections, the process of making violence, and hence the bullies, less attractive is a consequence of enhancing the attractiveness of students who support the victim and reject violent attitudes. Both processes run in parallel and are complementary; the more they like “brave” children, the less they like violent ones.

They were in the playground, and a boy from the other class said, “go get A,” and some joined and chased him and threw him to the ground, kicking him. Those who did not know about it were shocked in class and expressed that those boys had behaved very cowardly. They said that they were not going to allow this attitude anymore. The class made them less attractive, and those who had joined the first boy were very embarrassed because no one was amused with their actions, and they looked ridiculous (…). On the other hand, two children had taken a stand in protecting him [the victim] and had told the others to stop. Well, these two children were highly praised, their appeal was enhanced, and they became an example of bravery [Teacher 5—School 4].

Consequently, children started to only choose “brave” peers as friends, as described by teachers from two schools involved in the study. They also said that students who were temporarily not a part of the *Zero Violence Brave Club* tried to change their behavior because they did not like to be considered cowards or to be separated from the group. Therefore, the transformation in children's preferences led to a change of behaviors and roles of certain students in the group, as a teacher reflected upon:

When they started the first year of primary education, the one who was the most successful in class was not the kindest child or the one expressing most solidarity, but the cockiest, the worst teaser, and who had more power over others. Little by little, the situation changed, and the most caring and solidary children became role models for others; they are much more valued. They were often looked at as an example of super brave children because they never left the club. That boy who was the cockiest even said that he wanted to be always courageous, and he was significantly affected when he was put out of the club. A boy dreamt that he wanted to stay in the club the whole year. Also with the girls, those who compete with each other have lost attractiveness, those who lie, who criticize others, who bother,… and there is a very kind girl who has gained confidence, many other girls want to play with her now because she is also seen as an example of bravery [Teacher 5—School 4].

### Creating Safe and Healthy Relationships From Childhood

The above analysis provides knowledge on how creating contexts during childhood based on the Zero Violence Brave Club contributes to preventing and overcoming bullying. This analysis allows us to study how the evidence-based aspects that this action integrates—such as the need to make existing violence visible, creating friendship and solidarity networks and emptying violence of its attractiveness—contribute to creating violence-free relationships. We want to conclude the section by showing an impact of the Zero Violence Brave Club identified by the teachers interviewed in the present study: how the peer groups create social environments that contribute to the emergence of healthy relationships, even where there were none before.

**Figure 2:**
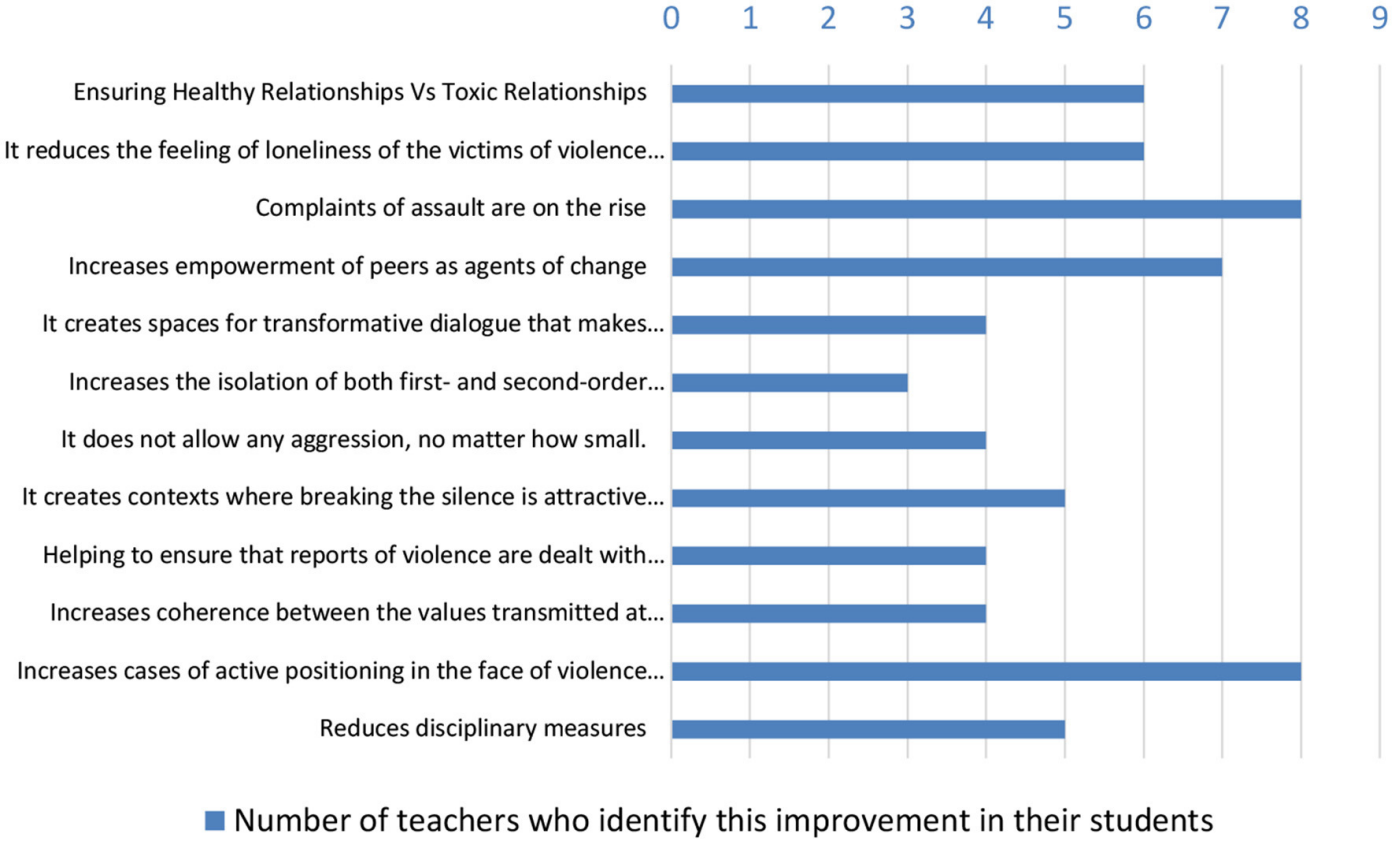
Impact of the Zero Violence Brave Club identified by teachers.

Figure 2 shows the overall results for 9 of the 10 teachers who responded to items 10, 12, and 13 of the online interview. As can be seen, eight of the nine teachers identified an increase in aggression reporting and increased active positioning in the face of violence thanks to the brave club. Therefore, in these nine classrooms, this action is achieving safer contexts, on the one hand, because it makes existing violence more visible and, on the other hand, because it reaches a protective response from their classmates when aggression occurs. A significant contribution of the Brave's Club in violence prevention in childhood is its impact on the children who are victims of violence and the other relationships within the group, in the form of healthier relationships. In this sense, factors that in different contexts could be risk factors for bullying, such as socially vulnerable children and children with specific learning difficulties, are transformed into opportunities to create safe and egalitarian relationships from childhood onward. The testimony of two teachers from different schools shows how these contexts of protection and transformation are emerging in the classroom:

Two years ago, a pupil with self-esteem problems, low frustration tolerance, difficulties for expressing himself and behavioral concerns at home did not like school. The creation of the brave club that same year, the possibility of speaking in these assemblies brought about a significant change in him. He realized that he had a space for dialogue, where he was going to be listened to if he had a problem, where if he made a mistake, nothing would happen because we were going to help him (…). Realizing that he could speak freely, tell what was happening to him, and be respected improved his self-esteem and behavior at home because he started to talk there too about what was happening to him and what he needed. His relationship with his parents changed (…) [Teacher 9—School 7].

One pupil with dyslexia, at the beginning of the fourth year, did not want to be in class; he dressed with a lot of covering, even with a scarf over his face. At 10 a.m. he already asked if it was still long before going home. As we worked with the brave club, he was able to uncover himself and participate. He showed the potential of the arguments he had “hidden under his scarf” (…). This year he was elected as a class delegate after daring to stand as a candidate. Now he plays with his classmates, and his mother says he looks like a whole new person [Teacher 3—School 2].

As shown in Figure 2, another of the most identified impacts (among seven of the nine teachers interviewed) is the increase in peer empowerment as agents of change for overcoming violence. This result is consistent with existing research. To break the law of silence that prevails in educational centers and protect aggressors, upstander environments are required, as well as people capable of protecting victims and all those who defend them (47). The Zero Violence Brave Club is transforming the relationships created between students and the coherence and active and supportive position that teachers have with their students. Knowing that the teaching staff, the school, has a clear and courageous role in the face of violence increases the chances that children will not be left alone in a bullying situation for years to come. In this sense, a teacher explains how they arrived in time to stop a case of school bullying in a child with whom the Zero Violence Brave Club had worked years before:

In another class, a boy was a doubling the year and had been doing braves club for 4 years. That year in his class, it was not done, but he sought help and dared to report the harassment that some classmates did to him outside the school. He told me about it 1 day when I passed by his house. They painted graffiti on the door of his house; they cheated him by exchanging video games with him that did not work; one boy even made a video on his YouTube channel insulting him. This pupil is a child with a family situation that makes him very vulnerable (…); the fact of having done the brave club gave him the strength to seek help and not put up with it [Teacher 6—School 4].

Finally, it is worth noting the impact that six of the nine teachers identified about the fact that the Brave Club creates healthy relationships instead of toxic ones and decreases the feeling of loneliness that traditionally surrounds victims of school bullying and those who stand up for them. One of the teachers commented on this.

In my third year of primary school students (8 or 9 years old) I see how they reject the bullying attitudes of some and they separate those relationships they find toxic from those they do want to have. And you can see it because if they have to deal with them daily because of some group work or something that a teacher tells them to do… they do it, but not afterwards, when they can choose [Teacher 2—School 1].

Nevertheless, barriers are also found. A teacher reported having a different experience in this regard, as the most popular students were still those with the worst behaviors. Other teachers coincide that these popular kids try to exercise power in the classroom to maintain their attractiveness among their peers. In this case, implementing the *Zero Violence Brave Club* becomes slower and more complicated, with the role of teachers being crucial to combat these barriers.

## Discussion

This research has analyzed the contribution of the *Zero Violence Brave Club* to preventing and overcoming violence at schools. The results corroborate the previous study findings in this field while providing novel insights on the actions and strategies to combat bullying in educational contexts. The study contributes to one of the main goals stated through the United Nations Sustainable Development Goals, precisely goal number 4: the shaping of quality education for all^2^.

Our study shows that the *Zero Violence Brave Club* is an effective strategy to improve cohesion and combat bullying in schools. All schools improved awareness about existing school violence, reduced violent behaviors, and developed more supportive and healthy relationships among students and with the overall community. Also, the fact that the schools were diverse in terms of size, type of location, public or private ownership, religious or lay background, and population served suggested that the impact of educational interventions does not depend on the context and that the strategy could be replicated in other contexts with similar benefits.

Second, three main characteristics of the *Zero Violence Brave Club* were identified that contributed to bullying prevention and reduction: (1) shedding light on the existing violence as a facilitator to breaking the silence on bullying cases; (2) the promotion of positive feelings of respect, solidarity, and friendship as protective factors in front of bullying; and (3) reducing the attractiveness of violence and increasing that of kindness. These results are consistent with previous research on strategies to combat violence in schools. Existing research has identified that creating spaces of dialogue helps break the silence about bullying by opening up communication channels where students with teachers or school staff discuss about the ways to ensure safety (59). In the *Zero Violence Brave Club*, these possibilities of dialogue occur and contribute to creating safe environments, enabling victims to denounce and support them, which are effective strategies identified in previous research (60). In this regard, teachers' efforts to foster peer support in the *Zero Violence Brave Club* encouraged children to speak up about conflict situations they had observed or experienced in the school and to be active against bullies. This evidence is in line with the extensive literature on the critical role of active bystanders, or upstanders, in stopping violence in schools and other related educational environments (31, 32).

Previous research also stated that educational programs based on socio-emotional learning foster a better coexistence (50). These programs focus on promoting children's learning of emotional skills such as empathy. The *Zero Violence Brave Club* has demonstrated to be effective in creating such feelings and in going further by encouraging students to practice respect, solidarity, and becoming friendlier. Also, they learned why it is essential to have good friends and freely choose their friends among those who treat them well. These strengthened friendships, in turn, enabled children to protect themselves from being bullied or harassed by their peers.

Previous research had also found that bullying behaviors are often associated with higher popularity and social reward from peers, which can contribute to sustaining such behaviors (35). The attractiveness of violent behaviors was also examined in research on the preventive socialization of gender violence among adolescents (39), which focused on the fact that tastes and desires defined in the socialization process can be changed through alternative interactions and dialogues (41). In this sense, research from socioneuroscience is also making progress concerning how specific social experiences can weaken, at a cognitive level, the dominant coercive discourse learned and consequently lead to greater freedom in relationships (61). The present study found that teachers' and children's participation in the *Zero Violence Brave Club* facilitated such transformations. Students who behaved aggressively were perceived as less attractive, leading to the change of their violent behavior.

Overall, the *Zero Violence Brave Club* is a comprehensive approach to counter and prevent bullying. It comprises several of the strategies shown in the literature as effective in addressing bullying and involves families and communities into agreeing about the school's norms and into improving school climate (20, 25, 27, 28). It leads to the whole community becoming active bystanders, or upstanders, improving coexistence and enhancing the possibilities of success.

Finally, our study provided evidence that the *Zero Violence Brave Club* contributed to improving children's mental health and psychological wellbeing, particularly bullying victims. We collected evidence regarding children feeling safer, less fearful, more confident, and empowered to explain violent episodes, to face aggressors, or to participate in certain activities that are indicators of psychological wellbeing. Furthermore, some of the impacts of this intervention were increased peer support, respect and solidarity, and an overall climate against violence, creating favorable conditions for a healthier environment. The scope of the study did not allow to reveal long-term impacts on children's mental health regarding the prevention of psychiatric disorders. However, as long as bullying has been associated with the development of mental health problems, the reduction and prevention of bullying is likely to contribute to reducing the probability that these problems appear.

Although the findings described above offer new insights to deepen into our understanding of bullying and how it can be overcome, the study has some limitations to be considered in future studies. Firstly, we selected a purposeful sample that cannot represent all the schools and teachers implementing the *Zero Violence Brave Club*. Both the schools and teachers were selected based on their experience in implementing the *Zero Violence Brave Club* and their knowledge of the programme to choose a sample that could provide the most relevant information on the object of study. Still, diversity in the selection of both the schools and teachers for this study has been ensured. Secondly, as researchers, we have been involved in the training, implementation, and assessment of the DMVP along our academic career, which could act as bias in the interpretation of the results. This experience also contributed to having a deeper understanding of the reality that has been studied. Finally, because of the qualitative nature of the research and the limited sample, the findings allow understanding the processes that make the *Zero Violence Brave Club* an effective strategy against bullying but are not to be generalized to other schools. Future research can further develop on the contributions of the *Zero Violence Brave Club* for preventing and overcoming bullying with a broader and more diverse sample. This strategy is already functioning in Latin American and European schools that are part of the international network of Schools as Learning Communities, so its implementation and impacts in diverse geographical contexts can be explored (43). When the study was designed, the schools had only been involved in the *Zero Violence Brave Club* programme for a short period. In this sense, we considered that teachers could identify the improvements that were taking place from the onset and where the implementation of this action needs to be improved. As data on the effects of the intervention on pupils and families are already available (38), the present research focuses on collecting the voice of teachers, being aware of its limitations in this regard. In the future, these limitations will be covered by additional research that reports information from more voices of the educational communities in the seven schools (children, relatives, and the overall community) to have a more complete and accurate understanding of the phenomenon explored, including family's perceptions about their role in bullying prevention strategies, the process of norm construction with the participation of the whole community, the impact of the *Zero Violence Brave Club* beyond the school setting, and the contributions to the mental health and wellbeing of other community members.

## Data Availability Statement

The raw data supporting the conclusions of this article will be made available by the authors, without undue reservation.

## Ethics Statement

The studies involving human participants were reviewed and approved by Ethics Board of the Community of Researchers on Excellence for All (CREA). The patients/participants provided their written informed consent to participate in this study.

## Author Contributions

ED and ER-C developed the conceptualization of the article. ER-C carried out data collection. All authors coordinated by OR, carried out data analysis. The manuscript was written by MR-S and revised by all other authors.

## Conflict of Interest

The authors declare that the research was conducted in the absence of any commercial or financial relationships that could be construed as a potential conflict of interest.

## Footnotes

^1^The Ethics Board was composed of Dr. Marta Soler (president), who has expertise in the evaluation of projects from the European Framework Programme of Research of the European Union and of the European projects in the area of ethics; Dr. Teresa Sordé, who has expertise in the evaluation of projects from the European Framework Programme of Research and is a researcher in the area of Roma studies; Dr. Patricia Melgar, a founding member of the Catalan Platform Against Gender Violence and a researcher in the area of gender and gender violence; Dr. Sandra Racionero, a former secretary and member of the Ethics Board at Loyola University Andalusia (2016–2018) and a review panel member for COST action proposals in the area of health; Dr. Cristina Pulido, an expert in data protection policies and child protection in research and communication and a researcher in communication studies; and Dr. Esther Oliver, who has expertise in the evaluation of projects from the European Framework Programme of Research and is a researcher in the area of gender violence.

^2^UN Sustainable Development Goals: https://www.un.org/sustainabledevelopment/
